# How does addressing diabetes distress lead to positive glycemic change? Results from the EMBARK trial

**DOI:** 10.1016/j.pec.2025.108748

**Published:** 2025-06-23

**Authors:** Lawrence Fisher, Susan Guzman, William H. Polonsky, Lisa Strycker, Katherine Greenberg, Danielle M. Hessler

**Affiliations:** a University of California, San Francisco, San Francisco, CA, United States; b Behavioral Diabetes Institute, San Diego, CA, United States; c Oregon Research Institute, Springfield, OR, United States

**Keywords:** Diabetes distress, Glycemic outcomes, Mechanisms of change

## Abstract

**Objective:**

To document the effectiveness of diabetes distress (DD) interventions and to suggest how the improvements in DD, documented in a previous report, led to improved glycemic outcomes.

**Methods:**

Individuals with T1D (*n* = 276) with elevated DD (>2 on T1 Diabetes Distress Scale) and HbA1c (>7.5 %) were assigned to: (1) StreamLine, a diabetes self-management program; (2) TunedIn, an ACT-based (Acceptance and Commitment Therapy), emotion-focused DD program; or (3) FixIt, an integration of Streamline and TunedIn. Previous research had shown reduced DD (Stage 1) and improved glycemic outcomes (Stage 4) from baseline to 12-months. Here we assess two intermediate stages: adoption of a new emotional perspective (Stage 2) and improvements in diabetes-related problem solving (Stage 3). A structural equation model was specified to evaluate a hypothesized, 4-stage model of change.

**Results:**

Improvements occurred in all three interventions at all 4 stages. TunedIn and FixIt, however, compared to StreamLine, displayed greater improvements in Stages 1 through 3; while StreamLine and TunedIn displayed greater improvements in glycemic outcomes than FixIt. Associations between reductions in DD and glycemic change were explained through improvements in emotional perspective taking and problem solving.

**Conclusion:**

Although all three intervention groups showed improvement, TunedIn demonstrated the most comprehensive benefits. Findings indicate how reductions in diabetes distress, documented in a previous report, are linked with improved glycemic outcomes through changes in emotional perspective-taking, which then allow for improved diabetes-related problem solving. This sequence can form the basis for designing effective intervention programs to reduce DD and improve overall diabetes management.

## 1. Introduction

Diabetes distress (DD) refers to the worries, fears and concerns that are part of living with and managing this demanding chronic disease over time [1]. Elevated DD is highly prevalent among adults with diabetes (75 % for T1D; 64 % for T2D) [2,3] and it has been linked with poor management (e.g., missed medication taking), lower quality of life and negative glycemic outcomes (TIR, HbA1c) [2,3]. Hence, DD remains a significant clinical problem.

Several narrative reviews and meta-analyses have documented several approaches to reducing elevated DD [4–6], including educational, behavioral, affective, and so-called third wave approaches that focus on acceptance and mindfulness [7–11]. These reviews suggest that DD is highly responsive to intervention, with the strongest and most consistent effects stemming from interventions that target the emotional side of diabetes directly, rather than focusing exclusively on education or disease management [12,13].

Theories of emotion regulation (ER) provide a useful conceptual platform for designing emotion-based interventions to reduce DD. ER identifies the strategies that people use to manage the variety of fears and worries they may have about diabetes [12,14,15]. Several models of ER, such as the Broaden-and-Build and the Dynamic Model of Affect, suggest that elevated DD and problematic ER act as “barriers” that prevent the use of more adaptive management decisions [11,16,17]. In these cases, the management decisions that people make are driven primarily by the urge to reduce the intensity of troubling affect. For example, some people with diabetes attempt to reduce feelings of hopelessness by avoiding self-management tasks that remind them of their feelings of powerlessness, lack of self-efficacy, or “failure” in diabetes. Thus, intense feelings block consideration of other, more adaptive problem-solving options [1,18].

ACT (Acceptance, Commitment Therapy) is an evidenced-based intervention strategy that has been successfully employed to improve ER among individuals with diabetes or other chronic conditions [11, 18–20]. Following ACT principles, DD is first reduced through a process of “diabetes-related emotional validation.” This refers to identifying the intensity and the specific sources of current DD, whereby the emotions involved are explicitly labelled (e.g., fear, frustration, powerlessness, shame) and the underlying diabetes management behaviors that are driven by these emotions are identified and normalized [21]. Normalization helps individuals accept the common frustrations of disease management and reduces self-criticism, guilt and blame. Once DD is both acknowledged and connected to emotion-driven behaviors, ACT focuses on enabling the individual to “adopt a different perspective.” As individuals learn to recognize and accept their fears and concerns, they can consider other management options, ones that are not driven primarily by emotions but, instead, are more in line with their values and goals [21]. This “simple” separation of feelings from behavior fosters a change in perspective that often surprises people. They begin to recognize that they do not have to be controlled by their feelings: they can still experience painful feelings, but they can act differently by considering other, far less dangerous problem-solving options.

The randomized, three-arm EMBARK study [13] was designed to evaluate interventions to reduce elevated DD and elevated HbA1c for adults with T1D. It tested the relative effectiveness of an ACT-based, emotion focused intervention (TunedIn) with an education/management intervention (StreamLine), and an intervention that combined TunedIn and StreamLine (FixIt). Twelve-month follow-up data showed that all three interventions led to significant reductions in both DD and HbA1c; but, overall, TunedIn, the emotion-focused intervention, showed the largest, most meaningful changes over time. This is particularly important because TunedIn was exclusively emotion-focused and did not include diabetes education or an emphasis on diabetes management. Instead, it focused on reducing DD to enable the adoption of a different emotion/management perspective to enhancing diabetes-related problem solving and improve glycemic outcomes. Hence, the longitudinal results from EMBARK demonstrated the overall value of a time-limited, emotion-focused, DD-reduction strategy for adults with T1D.

**Fig. 1.**
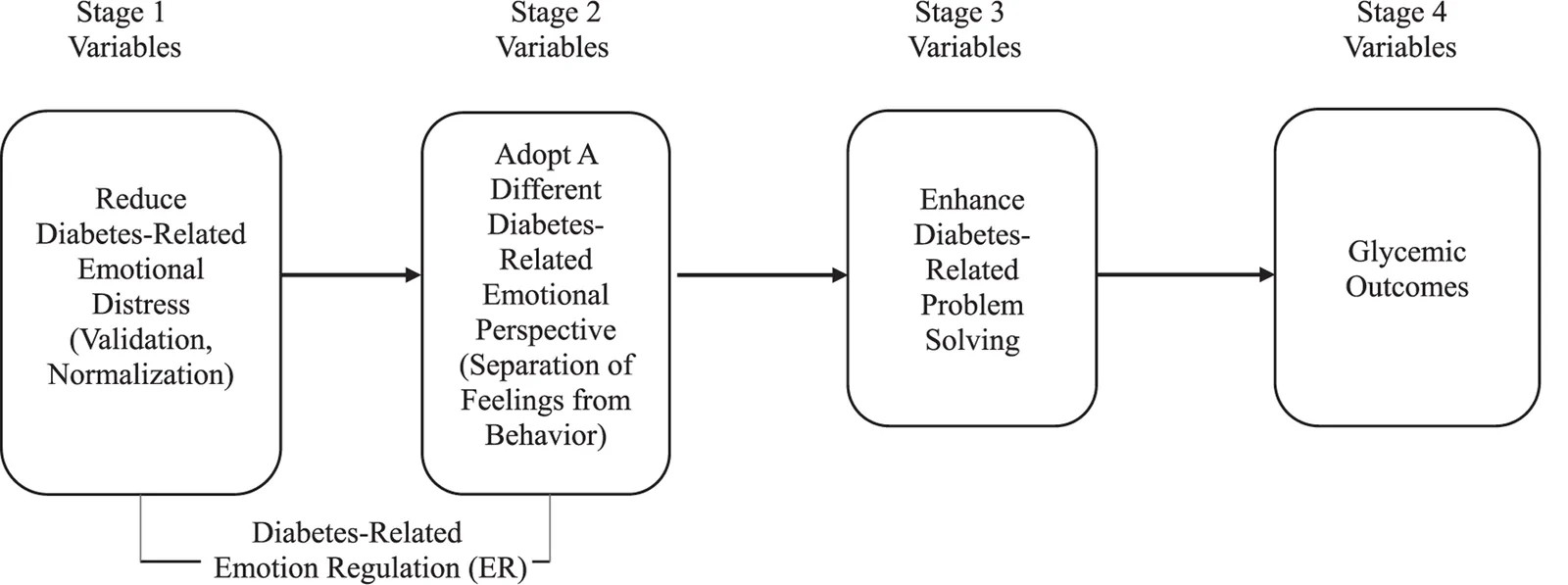
A four-stage hypothesized model of how baseline to 12-month changes in diabetes-related ER are associated with changes in diabetes-related outcomes.

The goal of the present report is to examine how reductions in DD were associated with improvements in glycemic outcomes in the EMBARK study. Analyses first documented baseline-to-12-month change in four sets of variables for the total sample and for each intervention group: reduction of DD, change in emotional perspective, improvement in diabetes-related problem solving, and improvement in glycemic outcomes (HbA1c, number of hypoglycemic episodes). Second, a structural equation model was specified with a linear sequence of hypothesized connections, guided by ACT and the barrier hypothesis, to evaluate the associations of baseline to 12-month change across the four groups of variables: from reductions in DD (Stage 1), to changes in emotional perspective (Stage 2), to improvements in diabetes-related problem solving (Stage 3), to changes in glycemic outcomes (Stage 4) (Fig. 1). Understanding how changes in diabetes-related ER are linked to improvements in problem-solving and glycemic outcomes are crucial to informing our understanding of the process of change and enhancing the design and structure of future DD reduction interventions.

## 2. Methods

### 2.1. Design and participants

The EMBARK recruitment procedure has been described previously [13]. Briefly, adults with T1D were recruited from the Taking Control Of Your Diabetes Research Registry and the T1D Exchange; and from several community clinics and diabetes organizations. Prospective participants were sent emails and/or social media posts and interested individuals were encouraged to respond by email or phone. Patients from diabetes clinics were informed that a member of the research team would contact them to tell them about the project unless they opted out by returning a project-supplied post-card or by calling a toll-free number. Upon contact, the project was explained, informed consent obtained, and the Type 1 Diabetes Distress Scale (T1-DDS) was administered [22]. Permission was then requested to obtain their most recent HbA1c results from their clinic. If none were available in the last 3 months, a pre-paid laboratory slip or home HbA1c test kit was mailed. Eligible participants then were asked to complete an online survey, for which they received a $40 gift card. The same online survey and lab test also were completed 12 months after baseline.

Inclusion criteria were: > 19 years of age; diagnosis of T1D for at least 12 months; ability to read, write, and speak English; a score of > 2.0 on the T1-DDS total score, indicating elevated DD [22], a recent HbA1c value > 7.5 %; no severe complications (end-stage renal disease, blindness); absence of psychosis or dementia; and Internet access.

Participants then were randomly assigned by the research coordinator (1:1:1 allocation), using a computer-generated protocol, to one of the three virtually delivered, group-based interventions containing 8–15 participants per group. To address concerns about including highly distressed participants in the study without intervention, a no-treatment control group was not included. Data were collected in 2019–2023, and analyzed in 2023–2024. All study activities were approved by the University of California, San Francisco Institutional Review Board and the trial was registered at clinicaltrials.gov (identifier NCT04016558).

### 2.2. Interventions

The three interventions have been described previously [13]. Streamline is a diabetes-education/management program featuring one 3-h virtual workshop followed by five individual phone calls. The workshop provided a brief diabetes review including proper basal insulin and bolus dosing. It also included a structured program to identify and resolve specific glucose challenges. Steps included organizing their “diabetes toolkit,” identifying a specific blood glucose problem, exploring the problem through pattern recognition, making a change, and collecting new data to see what happened.

TunedIn addressed the emotional side of diabetes. It featured two 3-h virtual group workshops, four 1-h group Zoom sessions, and a brief phone call with the group leader. The workshops provided information about DD, reviewed each participant’s DD profile from the T1-DDS, and included a structured program to reduce DD, following the ACT principles described above.

FixIt was a modified combination of Streamline and TunedIn. The TunedIn portion featured two 3-h workshops, three 1-h group Zoom calls, and one 10-min phone call. The subsequent Streamline portion included one 3-h workshop and four phone calls. At the end, group leaders delivered two, 1-h virtual Zoom sessions, integrating TunedIn and Streamline.

### 2.3. Measures

Participant data were collected on age, gender identity, ethnicity and racial identity, years with T1D, education, number of complications from a list of 11, and use of CGM (yes/no) and insulin pump (yes/no).

Four sets of variables were assessed, corresponding to the four stages of the hypothesized model, based on theoretical relevance and use in the literature. Stage 1: the intensity and source(s) of DD were identified by the T1-Diabetes Distress Scale (T1DDS). The T1-DDS [2]contains 28 items. The total score (alpha=.84) is based on the mean of the items included in 7 common “sources” of DD: management, powerlessness, hypoglycemia, eating, friends/family, physician, and negative social perceptions. Items are rated on a Likert scale from 1 (“not a problem”) to 6 (“a very serious problem”).

Stage 2: three measures were included to adequately examine changes in perspective taking. Two subscales were drawn from The Five Facet Mindfulness Questionnaire [23]. The 8-item Non-Judging Of Inner Experience (NonJudgeIE) scale assesses acceptance of feelings without judgement or self-criticism (alpha=.81). The 7-item Non-Reacting To Inner Experience Scale (NonReactIE) reflects an awareness of feelings and an ability to separate feelings from actions (alpha=.86). Items are rated on a Likert scale from 1 (“never or rarely true of me”) to 5 (“very often or very true of me”). Also included was a measure of diabetes-specific self-compassion: the 6-item Self Compassion Scale (SelfCom) [24] (alpha=.94). Items are rated on a Likert scale from 1 (“almost never”) to 5 (“almost always”).

Stage 3: Diabetes-Specific Problem Solving was assessed by 2 subscales from the Diabetes-Related Problem-Solving Scale [25]: Effective Problem Solving (EffectPS) (9 items; alpha=.76) and Avoidant Problem Solving (AvoidPS) (7 items; alpha=.75). Items in both are scored on a Likert scale from 1 (“not at all like me”) 5 (“extremely true of me”).

Stage 4: two measures were used to assess glycemic outcomes. HbA1c, recorded within the last three months, was assessed from clinic records, a home test kit, or a prepaid lab slip to a local clinic. Second was a single question regarding the number of self-reported hypoglycemic episodes (HYPO) < 70 mg/dL in the last week [26].

### 2.4. Statistical analyses

All analyses were carried out with SPSS (IBM Corp., 2023, Version 29.0.2.0) and Mplus (Muthén & Muthén, 1998–2017, Version 8.9) software. Descriptive statistics were computed to ensure that their distributions met normality assumptions. χ2 or student t tests were used to compare the three treatment conditions on baseline values of the variables of interest. Paired *t* tests and analysis of variance (ANOVA) were employed for the total sample and separately for each group to determine whether variables significantly changed from baseline to 12 months: the difference score was specified as the outcome and treatment group was a fixed effect. Initially, analyses controlled for demographic characteristics and disease status. Because none had a significant effect, they were excluded in the final models to avoid confounding. We also tested if differences occurred over the years of data collection – 2019–2023. None were observed, so date of data collection was not included in subsequent models. To aid interpretation of ANOVA results, Cohen’s *d* effect sizes were calculated (the absolute difference between baseline and 12-month means divided by the pooled standard deviation) and interpreted as follows: small effect *d* = 0.20, medium effect *d* = 0.50 and large effect *d* > 0.80. When a treatment group effect was statistically significant (*p* < .05), Fisher’s post hoc least significant difference procedure was used to determine if groups differed from each other. Comparisons of DD and HbA1c for the total sample and for each group have been reported in detail elsewhere [13] and are included herein for completeness of presentation.

Structural equation models were specified to examine the associations among baseline to 12-month change in a hypothesized linear sequence from Stages 1 through 4. Mplus uses an Expectation Maximization algorithm that allows for the handling of missing data, enabling the models to include all participants having at least one data point. Participant-level covariates of age, gender, use of continuous glucose monitoring, and number of health complications were initially included; however, these covariates had little or no significant effect and were excluded in parsimonious final models. To examine change, 12-month values of all variables were regressed on their respective baseline values. Regression parameters, covariances, means, and variances were estimated to determine relations among the 12-month variables in a progression across the stages of the model, while accounting for change in these variables from baseline to 12 months. Regression coefficients (B) were standardized based on the variance of other observed variables. Inspection of Mplus modification indices indicated that inclusion of an additional regression path that skipped a stage of the model would significantly improve model fit; thus, the regression of AvoidPS on DD was added.

## 3. Results

### 3.1. Participant characteristics

Details of the sample and the CONSORT diagram have been presented elsewhere [13]. Briefly, the sample included 276 adults: 97 in Streamline, 91 in TunedIn, and 88 in FixIt. Retention at 12 months was 81 %, with no significant group differences. Compared with those who dropped out (n = 51), those who completed the study (n = 225) reported lower mean baseline HbA1c values (8.2 % vs. 8.6 %; P = 0.02) and fewer years living with diabetes (22.98 vs. 27.89 years; P = 0.04). Mean (SD) age was 46.8 (15.1) years, 79.6 % identified as female, and mean (SD) baseline HbA1c was 8.3 % (0.9 %) (67.0 [9.8] mmol/mol). At baseline Streamline participants reported somewhat longer diabetes duration than those in the other groups (Table 1).

### 3.2. Changes in DD (Stage 1)

As reported previously [13], large, significant reductions in mean total DD from baseline to 12 months were found for the full sample and for each of the three intervention groups, with a significant treatment effect (*F(df*=*2)* = 5.85; *p* = .03) (Table 2). Notably, TunedIn (*d* = 1.14) and FixIt (*d* = 1.06) participants achieved a significantly greater reduction in DD than StreamLine participants (*d* = 0.58).

### 3.3. Changes in adopting a different emotional perspective (Stage 2)

Significant improvements for the total sample and for each intervention group occurred for NonJudgeIE (StreamLine *d* =.30, TunedIn *d* =.71, FixIt *d* =.67); improvements did not differ significantly by group (treatment effect (*F*(*df*=2) = 2.19, *p* = .12). There was a significant improvement for NonReactIE for the entire sample (*F*(*df*=2) = 8.72, *p* = .001) and for TunedIn (*d* = 0.57) and FixIt (*d* = 0.88), but not for StreamLine (*d* = 0.11). Similarly, a significant improvement also occurred for the entire sample for SelfCom (*F* = 5.96, *p* = .003), with TunedIn (*d* = 0.95) and FixIt (*d* = 0.96) yielding significantly greater improvement than StreamLine (*d* = 0.45). Overall, significant improvements were found in all three Stage 2 variables for the entire sample, with significantly greater change for TunedIn and FixIt than for StreamLine.

### 3.4. Changes in diabetes-related problem solving (Stage 3)

Significant improvements in both EffectPS (*F(df*=*2)* = 3.46, *p* = .034) and AvoidPS (*F* = 6.05, *p* = .003) occurred for the full sample, and for two of the three intervention groups: TunedIn (EffectPS *d* =0.62; AvoidPS *d* =0.39) and FixIt (EffectPS *d* = 0.41; AvoidPS *d* = 0.75). Change for StreamLine was nonsignificant (EffectPS *d* = 0.19; AvoidPS *d* = 0.10).

### 3.5. Changes in glycemic outcomes (Stage 4)

As previously reported [13] significant reductions in HbA1c occurred for the entire sample, and for each group separately, with significant differences among groups (treatment effect *F(df*=*2)* = 60.06, *p*.= .001). Greater reductions were found for StreamLine (*d* =0.80) and TunedIn (*d* = 0.75) than for FixIt (*d* = 0.24). The sample as a whole also showed modest reductions in HYPO over time, but these changes were statistically nonsignificant and change did not differ by group (treatment effect *F(df*=*2)* = 0.94, *p* = .39).

### 3.6. Evaluation of the specified path model

**Fig. 2.**
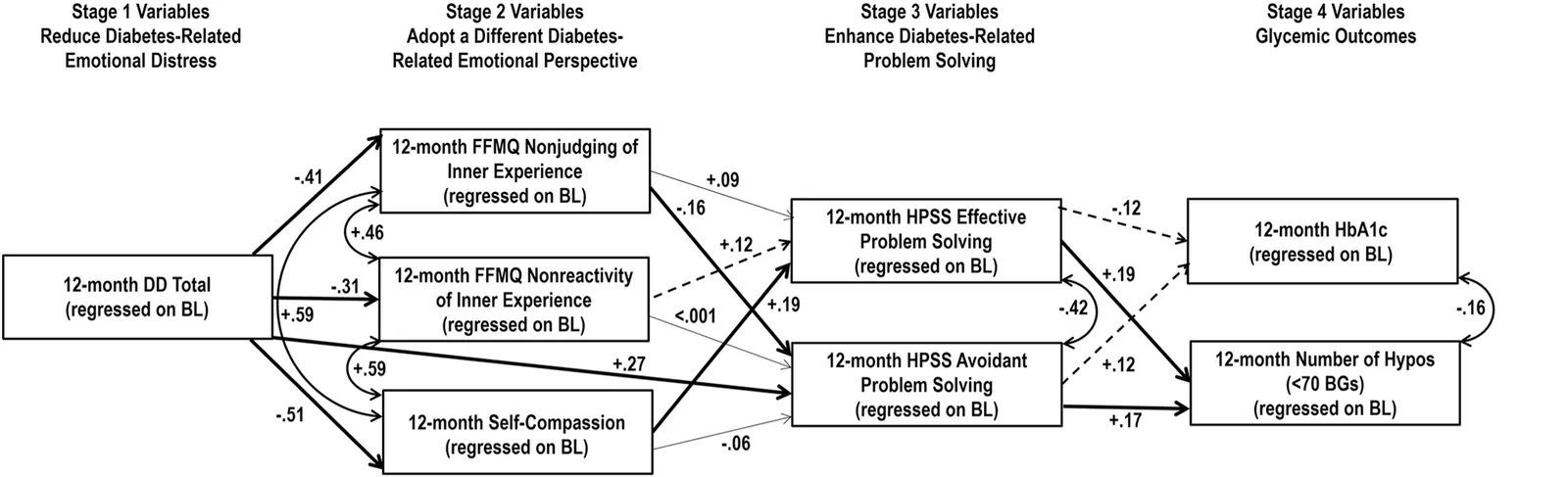
Structural equation path model results with standardized regression coefficients. Note: The diagram includes all paths specified in the model. Thick solid lines indicate paths with p levels < .05; dotted lines indicate p levels.05 to < .09; and thin solid lines indicate paths with p levels > .09.

The structural equation path model with standardized regression coefficients is shown in Fig. 2 (regression estimates are presented in Appendix A). The fit indices indicated a good fit of the model to the data (χ^2^(*df* = 65) = 119.85, *p* < .0001, CFI = .916, TLI = .910, RMSEA = .055, SRMR = .049). (Error variance was estimated for all variables in the model, but are not shown in Fig. 2 to simplify the presentation.) Using the baseline to 12-month change scores, a review of the independent paths across the four stages indicated that reductions in diabetes distress (Stage 1) were associated with improvements in NonJudgeIE, NonReactIE and SelfCom (Stage 2), which were associated with improvements in both EffectPS and AvoidPS diabetes-related problem solving (Stage 3), which, in turn, were nominally associated with reductions in HbA1c, and with significant increases in HYPO (Stage 4). The latter finding was unexpected, but understandable. As reflected by the negative association between HbA1c and HYPO, reductions in HbA1c from elevated baseline levels were associated with modest increases in hypoglycemic episodes.

Several aspects of path model results displayed in Fig. 2 are noteworthy. First, all associations were in the expected direction, and improvements in one stage were associated with improvements in the next stage in the expected sequence. Second, only one significant path skipped a stage: reductions in DD (Stage 1) were directly associated with reductions in AvoidPS (Stage 3) without operating through Stage 2. In all other cases the significant pathways followed the expected sequence. Third, all variables within a stage of the model were significantly interrelated, which adds to the model’s coherence. Fourth, when participant characteristics were included in the mode, they were not significantly related to the variables of interest, and therefore were excluded in the final model. Fifth, when intervention group was added to the model, the resulting associations did not differ by group, suggesting the sequence of change did not vary by intervention group, and the model fit was not enhanced (χ^2^(*df* = 93) = 740.33, *p* < .0001, CFI = .570, TLI = .371, RMSEA = .159, SRMR = .180).

Supplementary analyses were conducted to test two plausible, alternative models. One model reversed the order of Stages 1 and 2 (χ^2^(*df* = 70) = 172.83, *p* < .0001, CFI = .880, TLI = .843, RMSEA = .073, SRMR = .067), and the other model reversed the order of Stages 2 and 3 (χ^2^(*df* = 65) = 197.31, *p* < .0001, CFI = .846, TLI = .782, RMSEA = .086, SRMR = .064). In both cases, the model fit indices indicated worse fit, and the regression estimates for the pathways between variables were inconsistent. Thus, the final model reflects the most parsimonious description of the hypothesized, underlying mechanisms of change between baseline and 12 months.

## 4. Discussion and conclusion

### 4.1. Discussion

Two key findings highlight the results. First, the emotion-focused interventions—TunedIn and FixIt—yielded significantly greater improvement in managing distress and improving diabetes-related problem-solving than StreamLine. Furthermore, TunedIn displayed significant improvements in HbA1c, similar to StreamLine, even though TunedIn was not focused on self-management. Taken together, these two findings suggest that a brief, targeted, DD-focused intervention, such as TunedIn, offers the strongest, most comprehensive benefits—more than an educational or combined emotion/educational intervention alone. It may be that a combined education/emotion intervention, like FixIt, is too complex and overwhelming for participants when delivered at the same time [13].

The second key finding is that, although other plausible alternative models were assessed, our hypothesized path model displays the best fit to the data. Furthermore, the model suggests how change may take place for the entire sample, regardless of study arm. While TunedIn participants reported the strongest and most comprehensive improvements, the mechanisms by which decreases in DD were linked to glycemic improvement operated similarly for all three interventions. Diabetes-related feelings, beliefs and expectations are powerful, they heavily impact management decisions and they set the stage for glycemic outcomes [27]. For example, worries and fears about hypoglycemic episodes can drive an individual to keep glucose levels high, despite excellent diabetes education and many years of self-management experience. Outcomes may improve, however, once these feelings, beliefs and expectations are addressed, labelled and normalized, once a different emotional perspective is adopted, and once more effective, value-based, diabetes-related problem-solving decisions occur.

In contrast to previous studies [12], the 4-stage model evaluated here is based on longitudinal, interventional change data, rather than on static, cross-sectional findings. Consequently, this parsimonious model efficiently illustrates at least some of the mechanisms involved and some of the processes of change that occurred with the EMBARK interventions. Furthermore, the results underscore the importance of addressing the emotional side of diabetes early in a treatment program; a critical first step for achieving positive changes in diabetes-related problem solving and glycemic outcomes. Once elevated DD-related emotional barriers are reduced, we note how often participants identified different management options on their own, without additional educational/management input. This suggests that diabetes-related fears and worries often prevent participants from acting on what they already know to be in their best interest.

Three other aspects of the path model findings are noteworthy. First, not every variable within one stage is related to every variable in the next stage. This suggests that the mechanisms of change are complex and that multiple factors may be operative. Second, one significant path skipped a stage: reductions in DD (Stage 1) were directly linked with reductions in AvoidPS (Stage 3), suggesting that just “validating” diabetes-related fears and worries may lead to reduced avoidance –a powerful intervention tool in its own right for many people with diabetes. Third, the associations illustrated in the model operate for the entire sample, for each intervention separately and across individuals with different demographic and disease-related characteristics. This increases confidence in the generalizability of the mechanisms involved. Although it may be helpful to customize emotion-focused interventions to meet the specific needs of adults with T1D of different ages, ethnicities, disease status, etc., the processes of change seem to be consistent and generalizable.

Several limitations should be noted. First, although tests for differences based on participant demographic and disease status characteristics were nonsignificant, the interventions need to be evaluated with broader samples. Second, only two, somewhat generic glycemic measures, HbA1c and HYPO, were included. Future research should examine the effects of DD-reduction interventions on a wide range of outcomes, e.g., Time in Range. Third, although path models can help understand the processes of change, they use correlational data in sequence and do not directly document causation. Fourth, the current findings are based on change across only two time-points. Reductions in DD may operate as both cause and consequence of changes in glycemic outcomes: thus, multiple time points are necessary to fully articulate patterns of change.

### 4.2. Conclusion

In overview, EMBARK demonstrates how reductions in DD following intervention may be linked to improved diabetes-related problem solving and glycemic outcomes. They suggest a specified linear sequence of mechanisms through which these processes may operate, which can enhance our understanding of the processes involved when designing effective intervention programs to reduce DD, enhance quality of life and improve overall diabetes management.

### 4.3. Practical implications

These findings have important implications for clinical care. First, they highlight the need to fully integrate the emotional side of diabetes within the context of diabetes education and care in line with current ADA guidelines [28]. Doing so orients clinical interventions to the specific worries and concerns of each individual with diabetes, thus providing more person-centered care. Second, given the high prevalence of elevated DD, the responsiveness of DD to intervention and the simplicity of ACT-based interventions, we suggest that, with support, such efforts can be undertaken successfully by diabetes clinicians [29, 30]. Because DD intervention does not require extensive behavioral health training, implementing brief, targeted training programs for clinicians can aid in the incorporation of DD-related interventions into clinical care. Third, programs similar to EMBARK, (e.g., T1-REDEEM [1]), have demonstrated the cost- effectiveness of a virtual, group-based DD-reduction program. The group-based format can aid in establishing a community to help normalize the struggles of diabetes management and to provide an accepting, cost-effective context for addressing DD.

## Figures and Tables

**Table 1:** Participant Characteristics at Baseline by Intervention Group (*N* = 276).

	All *M* (*SD*) or % *N* = 276	Streamline *M* (*SD*) or % *n* = 97	TunedIn *M* (*SD*) or % *n* = 91	FixIt *M* (*SD*) or % *n* = 88	*F or χ^2^* (*p*)
Age (years)	46.81 (15.11)	48.88 (15.44)	47.34 (15.38)	43.99 (14.17)	2.52 (.082)
Diabetes Duration (years)	26.99 (15.30)	30.44 (15.47)	25.90 (14.74)	24.30 (15.15)	4.16 (.017)
Insulin Pump (% yes)	72.8% (201)	73.2% (71)	72.5% (66)	72.7% (64)	0.01 (.994)
CGM (% yes)	78.6% (217)	79.4% (77)	80.2% (73)	76.1% (67)	0.50 (.781)
Highest education level achieved (%)					11.97 (.063)
High school degree	2.6% (7)	1.5% (4)	0.4% (1)	0.7% (2)	
Some college	25.5% (70)	26.8% (26)	27.5% (25)	22.1% (19)	
Completed college	41.2% (113)	36.1% (35)	34.1% (31)	54.7% (47)	
Graduate school	30.7% (84	33.0% (32)	37.4% (34)	20.9% (18)	
Gender (%)					1.67 (.434)
Male	52 (18.9%)	22 (22.7%)	16 (17.6%)	14 (16.1%)	
Female	219 (79.6%)	74 (76.3%)	72 (79.1%)	73 (83.9%)	
FTM	3 (1.1%)	1 (1%)	2 (2.2%)	0 (0%)	
MTF	1 (0.4%)	0 (0%)	1 (1.1%)	0 (0%)	
Ethnicity (%)					0.84 (.657)
Native American	0.4% (1)	1.0% (1)	0% (0)	0% (0)	
Asian	1.1% (3)	2.1% (2)	0% (0)	1.1% (1)	
Native Hawaiian	0% (0)	0% (0)	0% (0)	0% (0)	
African American	2.9% (8)	1% (1)	2.2% (2)	5.7% (5)	
White	88.7% (244)	87.6% (85)	91.2% (83)	87.4% (76)	
>1 Race	5.1% (14)	5.2% (5)	5.5% (5)	4.6% (4)	
Hispanic/Latino (%)	10.4% (28)	11.7% (11)	12.2% (11)	7.0% (6)	1.58 (.455)
# Complications	3.32 (2.57)	3.43 (2.67)	3.35 (2.49)	3.16 (2.57)	0.27 (.762)
Total T1-DDS score	2.84 (0.76)	2.79 (0.83)	2.78 (0.73)	2.96 (0.73))	1.55 (.214)
HbA1c (mean %)	8.27 (0.93)	8.24 (0.86)	8.24 (0.87)	8.34 (1.07)	0.33 (.719)
HbA1c (mmol/mol)	67 (10.2)	67 (9.4)	67 (9.5)	68 (11.7)	0.33 (.719)

**Table 2:** Mean and effect size (*d*) for total sample and for each intervention group on secondary variables between baseline and 12-months (N = 276).

Scale	All: Mean baseline-12 months (*d*)	Streamline: Mean baseline-12 months (*d*)	TunedIn: Mean baseline-12 months (*d*)	FixIt: Mean baseline-12 months (*d*)	Between-Group Treatment Effect F (p)
Level 1: Diabetes Distress	**2.84–2.15** (.88)	**2.79–2.29** (.58) *1, 2*	**2.78–1.99** (1.14) *2*	**2.96–2.16** (1.06) *1*	5.85 (.0333)
Level 2: A New Perspective					
Non judging of inner experience (NonJudgeIE)	**27.65–31.30** (.53)	**28.99–31.29** (.30)	**27.93–32.13** (.71)	**25.84–30.46** (.67)	**2.19** (.12)
Non-reacting to inner experience (NonReactIE)	**21.54–24.07** (.49)	**23.09–23.68**(.11) *1, 2*	**21.35–24.34** (.57) *1*	**19.96–24.22** (.88) *2*	**8.72** (.001)
Self-Compassion (SelfCom)	**2.82–3.48** (.25)	**3.04–3.42** (.45) *1, 2*	**2.82–3.60** (.95) *1*	**2.56–3.44** (.96) *2*	5.96 (.003)
Level 3: Diabetes-Related Problem Solving					
Effective problem solving (EffectPS)	**18.03–20.50** (.40)	18.99–20.17 (.19) *1*	**17.69–21.42** (.62) *1*	**17.31–19.90** (.41)	3.45 (.034)
Avoidant problem solving (AvoidPS)	**7.19–5.21** (.42)	5.68–5.19 (.10) *1,2*	**7.14–4.94** (.39) *1*	**8.94–5.51** (.75) *2*	6.05 (.003)
Level 4: Glycemic Outcomes					
HbA1C	**8.18–7.68** (.60)	**8.17–7.52** (.80) *1*	**8.20–7.61** (.75) *2*	**8.16–7.94** (.24) *1,2*	60.06 (.001)
Number of hypoglycemic episodes (HYPO)	4.48–4.35 (.05)	4.58–4.80 (.09)	4.83–4.39 (.17)	4.00–3.78 (.08)	0.94 (.39)

NOTE: Bolded numbers indicate a significant change (<.05) between baseline and 12-months. Single numbers in italics *below* the change numbers indicate a significant difference (<.05) between that group and another group sharing the same number. Non-bolded numbers in parentheses next to the group difference numbers indicate the effect size of the change (the absolute difference between the baseline and 12-month numbers divided by the pooled standard deviation). These are interpreted as: small effect *d* =.20, medium effect *d* =.50 and large effect *d* =.80.
